# High body mass index increases the risk for prostate cancer and high Gleason score in northern Tanzania: data from prostate cancer screening

**DOI:** 10.3332/ecancer.2025.1898

**Published:** 2025-04-23

**Authors:** Bartholomeo Nicholaus Ngowi, Alex Mremi, Mshangama Seif, Frank Bright, Godrule Lyimo, Innocent Uggh, Modesta Paschal Mitao, Obadia Nyongole, Orgeness Jasper Mbwambo, Harcharan Gill, Mramba Nyindo, Kien Alfred Mteta, Blandina Theophil Mmbaga

**Affiliations:** 1 Faculty of Medicine, Kilimanjaro Christian Medical University College, PO Box 2240, Moshi, Tanzania; 2 Department of Urology, Kilimanjaro Christian Medical Centre, PO Box 3010, Moshi, Tanzania; 3 Kilimanjaro Clinical Research Institute, PO Box 2236, Moshi, Tanzania; 4 Department of Pathology, Kilimanjaro Christian Medical Centre, PO Box 3010, Moshi, Tanzania; 5 Department of Surgery, Muhimbili University of Health and Allied Sciences, PO Box 64001, Dar es Salaam, Tanzania; 6 Department of Urology and Surgery, Stanford University School of Medicine, Stanford, CA 94305, USA; † Deceased

**Keywords:** prostate cancer, body mass index, high Gleason Score, Tanzania

## Abstract

**Background:**

The association between body mass index (BMI) and prostate cancer (Pca) remains a controversial subject. Despite Pca being common in Tanzania, there is a scarcity of data on the role of BMI on Pca risk. This study aimed to assess the association between Pca and BMI among African men in Tanzania.

**Methods:**

This analysis included Tanzanian men aged ≥40 years who underwent prostate biopsy for the elevated prostate-specific antigen of >4 ng/mL during community-based Pca screening. Gleason scores of all Pca cases were determined by an experienced pathologist. BMI was calculated by dividing weight in kilograms by height in metre square. Participants were categorised into four BMI categories as follows: underweight (<18.5 kg/m^2^), normal weight (18.5–24.9 kg/m^2^), overweight (25.0–29.9 kg/m^2^) and obese (≥30 kg/m^2^). A high Gleason score refers to any score of ≥4+3. Logistic regression was used to estimate the odd ratio of each BMI category for the risk of Pca and high Gleason score.

**Results:**

A total of 572 men underwent prostate biopsy after being found to have an elevated prostate-specific antigen of >4 ng/mL during screening. Of these, normal BMI accounted for 233 (40.7%), while overweight and obesity accounted for 153 (26.7%) and 141 (24.7%), respectively. In multivariate analysis, overweight men had significantly higher odds of being diagnosed with Pca (OR 6.95, 95% CI; 3.43–14.06) as compared to their normal-weight counterparts. The strength of the association became stronger among obese participants (OR 23.65, 95%CI; 11.45–48.87). Similarly, there was a significant increase in the odds of being diagnosed with high Gleason score Pca among obese men (OR 3.63, 95%CI; 1.52–8.70).

**Conclusion:**

Tanzanian men with elevated prostate-specific antigen and a high BMI have a significant risk of being diagnosed with Pca, mostly with a high Gleason score. Normal BMI maintenance might help in reducing the risk of developing Pca.

## Introduction

Globally, prostate cancer (Pca) is the second most common cancer in men, accounting for about 15% of all men cancers [1–3]. Pca is the sixth most common cause of cancer death in the world [1, 2]. In Tanzania, Pca is the most common cancer in men, with a prevalence of up to 40% [4] and ranks the third leading cause of cancer death after oesophagus and liver cancers [5].

Apart from age, race and genetics as risk factors for Pca [6], recent studies have shown that obesity, as described by body mass index (BMI), is associated with a high chance of Pca diagnosis [7]. Additionally, the risk of dying from Pca is higher for men with high BMI as compared to their normal BMI counterparts [8]. Obesity has been implicated in the pathogenesis of several other cancers, including lung and oesophageal [1, 8, 10]. Obesity contributes to the progression of Pca through alteration in the endocrine system, specifically oestrogen and testosterone levels and insulin-like growth factor-1 [6, 10].

Similarly, obesity has also been associated with the Pca Gleason score [11]. Gleason score is an important prognostic factor representing the degree of aggressiveness of Pca based on architectural patterns [10]. The Gleason score ranges from 2 to 10, with high scores representing more aggressive disease or a poorer prognosis [10]. While several studies have shown a positive correlation between obesity [10–12] and a high Gleason score or high-grade Pca (HGPca), others have found no association [1, 6, 7].

Obesity is an epidemic global challenge, with a prevalence that is increasing as a result of a sedentary lifestyle that is associated with urbanisation and westernisation [9]. In the US, the prevalence of obesity has increased from 30%–42% from 1990 to 2018 [13]. The overall prevalence of obesity in Sub-Saharan Africa is 27%, where males account for 22.6% [9]. Tanzania is not left aside, with an obesity prevalence of up to 40% [14].

Studies of obesity in association with cancer, especially Pca, particularly in Tanzania are limited. Furthermore, previous studies on the association between obesity and Pca resulted in contradictory findings. The current study aimed to ascertain if obesity is related to Pca diagnosis and/or a high Gleason score among Tanzanian men, where both obesity and Pca are common.

## Material and methods

This study utilised data that were collected during a community-based Pca screening conducted in four administrative regions of the northern zone of Tanzania from May to September 2022.

The detailed methodology of the community-based Pca screening that included African men aged ≥40 years is available elsewhere [15]. Briefly, 3 districts were randomly selected from each of the 3 regions, namely Arusha, Manyara and Tanga, while all 7 districts of the Kilimanjaro region were included in the study making a total of 16 districts. The reason for including all seven districts of the Kilimanjaro region was the fact that this region accounts for >70% of Pca cases in the northern zone of Tanzania [16]. One health facility was selected from each of the selected districts and acted as the data collection center for the study. Study participants were invited to the study through various public announcement methods, such as social media, churches and mosques.

Consented participants were enquired about their social-demographic data, such as age, residence, marital status, education level as well as occupation. Anthropometric measurements were captured by trained study personnel as part of the screening.

The weight (in kg) and height (in cm) of each study participant were measured by weighing scale and stadiometer, respectively. BMI was calculated as weight divided by height in meters squared (kg/m^2^). Five milliliters of venous blood were collected in non-EDTA tubes from each study participant for PSA determination. The blood samples were analysed at the Kilimanjaro Clinical Research Institute’s (KCRI) biotechnology laboratory. A transabdominal ultrasound was also done at the time of Pca screening to determine the echogenicity and size of the prostate. A true cut biopsy (TCB) of the prostate was performed among participants who had PSA>4 ng/mL. The prostate tissue biopsies were sent to the Kilimanjaro Christian Medical Centre (KCMC) pathology department for processing and interpretation. Two independent pathologists reviewed the histological slides for quality assurance and consistency. All the collected data were kept in a pre-tested structured data extraction sheet.

### Data analysis

Statistical analyses were performed using STATA version 17.0 software. Pearson’s chi-squared test for categorical variables and the Kruskal–Wallis test for continuous variables were used to describe the relationship between variables. Logistic regression was applied to examine the strength and the direction of association between BMI and Pca detection. Similarly, the association between BMI and detection of HGPca was estimated using the same model, where HGPca was defined as a score of ≥4+3 [17]. Age, prostate volume, PSA, hypoechoic area and digital rectal examination (DRE) findings were adjusted for in the multivariate analysis. A *p* value <0.05 was considered to indicate statistical significance. BMI was categorised as follows: underweight, normal BMI, overweight and obese, <18.5, 18.5–24.9, 25.0–29.9 and ³30 kg/m^2^, respectively [9, 17]. Age was assessed as a categorical variable: 40–50, 51–60, 61–70 and >70 years. PSA and prostate volume were analysed after a logarithmic transformation.

## Results

### Characteristics of study participants

A total of 581 men aged **≥**40 years underwent TCB of the prostate, 9 (1.5%) had missing records of their height and weight, hence being excluded from this analysis. Of the 572 (98.5%) men who were analysed, 253 (44.2%) were aged **>**70 years with a median age of 70 (68–75) years. A total of 233 (40.7%)) participants had a normal BMI while overweight and obese accounted for 153 (26.7%) and 141 (24.7%), respectively. Of the 176 men who were confirmed histologically to have Pca, 79 (44.9%) had HGPca (Table 1).

### Characteristics of study participants stratified by their BMI

Factors such as PSA, abnormal DRE, Pca and HGPca varied significantly across the BMI categories. Obese men were older, with a median age of 72 (65–76) years and a lower median (SD) serum PSA of 6.8 (4.7–15.4) ng/mL as compared to other BMI categories. About half 92 (52.2%) of the participants detected with Pca were obese. While the lowest rate of HGPca was recorded among underweights (8 (10.1%)), the highest rate was found among obese (28 (35.4%)) (Table 2).

### Linear regression analysis on the association between BMI and Pca

In crude analysis, there was a significant increase in the odds of Pca detection with an increase in BMI. Underweight, overweight and obese participants had 3.21, 6.32 and 21.14 times higher odds of being diagnosed with Pca compared to participants with a normal BMI, respectively. After adjusting for age and PSA, obese participants had a slight reduction in the odds of Pca detection while overweight participants had a slight increase. The higher chance of Pca detection among underweight participants diminished following adjustment for age and PSA. In multivariate analysis, overweight and obese participants maintained significantly higher odds of Pca detection whereby, overweight and obese participants had 6.95 and 23.65 times higher odds for Pca detection as compared to the control group, respectively. In this model, the effect of BMI on Pca detection among the underweight group diminished (OR 1.01, 95%CI; 0.21–4.83). When BMI was incorporated into the multivariate models as a continuous variable, there were still significant positive associations between BMI and Pca detection (Table 3).

### Linear regression analysis on the association between BMI and HGPca

In crude analysis, participants who were obese had 3.95 times higher odds of having HGPca compared to participants with a normal BMI (95% CI; 1.80–8.69). After adjusting for age and PSA, obese participants had a slight reduction in the odds of HGPca detection. In multivariate analysis, participants who were obese had 3.63 times higher odds of having HGPca compared to participants with normal BMI (95% CI; 1.52–8.70) (Table 3).

## Discussion

The development of modern society, urbanisation and improvement of living standards are accompanied by an increased incidence of obesity as measured by BMI [1]. An increase in BMI has been linked to the development and progression of various cancers, including Pca [11]. The association between BMI and cancer, particularly Pca is a global health concern [7]. The current study assessed the relationship between BMI, Pca detection rate and Gleason score among African men in Tanzania aged ≥40 years with elevated serum PSA. The study indicated that men with elevated PSA and high BMI (overweight and obesity) are at increased risk of Pca, particularly a high-grade disease (high Gleason score).

The positive association between BMI and Pca in the current study is in line with similar other studies in Africa [8] and elsewhere [7, 17]. The exact mechanisms by which BMI brings about Pca are not very clear, but it is thought that a high BMI provides a conducive biological microenvironment for cancer onset and growth [7, 11]. Men with a high BMI produce high levels of adipokines and inflammatory cytokines, which are responsible for cancer growth. In addition, the low-grade inflammation associated with obesity causes metabolic changes such as insulin resistance, which leads to increased circulating insulin and IGF-1 levels [1, 7, 11]. Both insulin and IGF-1 promote carcinogenesis as well as halting apoptosis [7, 11, 17].

Contrarily, other investigators have found no association between BMI and Pca [12, 1], and others have reported a reduced risk of Pca among men with high BMI [8, 18]. The discrepancy could be explained by differences in the study population. Certain populations especially some of Asians, have diets rich in phytoestrogens, which tend to inhibit proliferation and exert a pro-apoptotic effect on the prostate and hence may reduce the risk of cancer formation even in the presence of obesity [11]. In the current study, participants volunteered to participate in the Pca screening, whereby only consented participants underwent TCB of the prostate due to elevated PSA (>4 ng/mL). These may have contributed to the observed difference by having a relatively large proportion of obese men 141 (24.7%). It is known that individual with high BMI are more concerned about their health and hence participate in screening as a result of their prior experience dealing with the associated hypertension, diabetes and hyperlipidaemia [8].

Compared to normal BMI, men with high BMI have a higher Pca mortality rate [8]. Nwadi *et al* [10] found a positive correlation between BMI and Gleason score among Nigerian men with Pca. Our finding is in line with several other studies done elsewhere that reported high BMI to be positively associated with an aggressive, lethal Pca based on the Gleason score [11, 12, 17]. The reason for this link could be due to the fact that men with high BMI create a microenvironment with low levels of testosterone and high levels of oestrogen that lead to the development of a more aggressive Pca [6, 7, 11]. In addition, individuals with high BMI have increased gene expression of the inflammatory transcript in the nuclear kappa beta pathway, which is responsible for enhancing tumour aggressiveness [11].

Contrary to our findings on the effect of BMI on the Gleason score, the study by Agalliu *et al* [19] reported that African men with aggressive Pca had a lower BMI compared to those with less aggressive disease [19]. This difference could be due to the fact that the previous study included hospital cases, which are likely to be symptomatic and have advanced cancer, hence disease related weight loss. Several other studies done elsewhere reported no association between BMI and HGPca [1, 6, 7]. The observed differences could be attributed to the differences in the study population and cancer screening practice, with the population in the present study being relatively unscreened and, therefore, expected to have high rates of aggressive disease.

In the current study, there was an inverse relationship between median PSA level and BMI. This observation is in line with other investigators who reported underweight men to have a higher mean serum PSA as compared to their obese counterparts [8]. Men with high BMI tend to have low levels of circulating PSA due to the effects of haemodilution [7, 9]. This highlights the need for careful interpretation of PSA in obese men.

### Strength and limitation

To the best of our knowledge, the current study is the first to report the relationship between obesity and Pca among Tanzanian men. Study participants were recruited through a community-based Pca screening representing all regions of the northern zone in Tanzania using the same screening protocol, and histology slides were reviewed by two independent pathologists for quality assurance and consistency.

Although our study findings were comparable to most other studies, the results should be considering the following limitations: first, the study was limited to African men who resided in Tanzania and, therefore, its relevance and generalisability among other African men in other parts of the world should be cautiously taken.

Second, the screening was done among participants who volunteered to participate in a community-based screening, and this might have subjected the study to volunteering bias.

## Conclusion

In this study of African men aged ≥40 years who were involved in a community-based Pca screening, overweight and obesity had a high rate of Pca detection, particularly HGPca. Maintaining a normal BMI might help in reducing the risk of developing Pca. Further research is needed to determine a possible causal relationship.

## Conflicts of interest

All listed authors have no conflict of interest to disclose.

## Consent for publication

Not applicable.

## Ethical approval and consent to participants

The ethical approval was obtained from the College Research Ethics Review Committee (CRERC) of Kilimanjaro Christian Medical University College (KCMUCo) with clearance number No. 2530. The informed consent was obtained from all participants before recruitment.

## Availability of data

Data will be available upon contacting corresponding author on reasonable request.

## Use of AI

AI was not used in the preparation of this work.

## Author contributions

Study design and conception: BNN, AM, OJM and BTM; data collection: BNN, AM, MS, JSM, FB, OJM and BTM; data analysis: BNN, MPM, GL and IU; interpretation of results: BNN, MPM and GL; drafting of the manuscript: BNN, AM and OJM; revision of manuscript: ON, HG, BTM and MN. All listed authors approved the manuscript.

## Figures and Tables

**Table 1.** Characteristics of men who underwent TCB of the prostate (*N* = 572).

Variable	*n*	%
Age (years)		
40–50	13	2.3
51–60	80	14.0
61–70	226	39.5
>70	253	44.2
Median age (IQR)	70 (63–75)	
BMI categories		
Underweight (<18.5)	45	7.9
Normal (18.5–24.9)	233	40.7
Overweight (25.0–29.9)	153	26.7
Obese (≥30)	141	24.7
PSA (ng/mL)		
4–10.0	330	57.7
10.001–20.00	109	19.1
>20	133	23.2
Median (IQR)	7.9 (5.38–17.68)	
Hypoechoic		
No	347	61.3
Yes	219	38.7
Pca		
No	396	69.3
Yes	176	30.7
Gleason score		
Low grade Pca	97	55.1
HGPca	79	44.9

**Table 2.** Characteristics of study participants stratified by BMI (*N* = 572).

		Underweight	Normal	Overweight	Obese	*p*-value
	**Total (%)**	**<18.5 kg/m^2^**	**18.5–24.9**	**25.0–29.9**	**≥ 30 kg/m^2^**	
No. patients (%)	572 (100%)	45(7.9%)	233 (40.7 %)	153 (26.7%)	141 (24.7%)	
Age (years)						0.090
40–50	13 (2.3%)	1 (7.7%)	7 (53.9%)	4 (30.8%)	1 (7.6%)	
51–60	80 (14.0%)	4 (5.0%)	38 (47.5%)	27 (33.8%)	11 (13.7%)	
61–70	226 (39.5%)	20 (8.8%)	86 (38.1%)	66 (29.2%)	54 (23.9%)	
>70	253 (44.2%)	20 (7.9%)	102 (40.3%)	56 (22.1%)	75 (29.7%)	
Median age (IQR)	70 (63–75)	70 (64–77)	70 (63–76)	68 (62–73)	72 (65–76)	
Abnormal DRE (yes)	253 (45.2%)	20 (7.9%)	90 (35.6%)	68 (26.9%)	75 (29.6%)	0.044
PSA (ng/mL)	7.9 (5.4–17.7)	15.5 (7.0–53.6)	7.7 (5.4–13.6)	7.4 (5.4–16.3)	6.8 (4.7–15.4)	<0.001
Prostate volume (mL)	28.7 (17.4–47.7)	29.7 (20.0–55.3)	27.6 (16.6–51.1)	29.6 (18.9–47.0)	27.8 (16.4–43.7)	0.506
Hypoechoic prostate (yes)	219 (38.6%)	21 (9.6%)	83 (37.9%)	54 (24.7%)	61 (27.8%)	0.253
Pca (yes) (%)	176/572 (30.7%)	10 (5.7%)	19 (10.8%)	55 (31.3%)	92 (52.2%)	<0.001
HGPca (yes)(%)	79 (44.8%)	8 (10.1%)	25 (31.7%)	18 (22.8%)	28 (35.4%)	0.006

**Table 3.** Odds ratio with 95% confidence interval for Pca detection and high Gleason according to BMI categories.

	Normal	Underweight	*p*-value	Overweight	*p*-value	Obese	*p*-value	Continuous BMI	*p*-value
Pca detection		OR (95% CI)		OR (95% CI)		OR (95% CI)			
Crude	Reference	3.21 (1.38–7.49)	<0.007	6.32 (3.56–11.21)	<0.001	21.14 (11.80–37.89)	<0.001	1.23 (1.18–1.29)	<0.001
Age-adjusted	Reference	3.08 (1.31–7.22)	<0.01	6.83 (3.81–12.23)	<0.001	20.98 (11.66–37.76)	<0.001	1.23 (1.18–1.29)	<0.001
PSA-adjusted	Reference	0.90 (0.19–4.20)	0.901	6.93 (3.49–13.76)	<0.001	20.43 (10.19–40.96)	<0.001	1.27 (1.20–1.35)	<0.001
Age and PSA- adjusted	Reference	0.91 (0.19–4.21)	0.907	7.00 (3.50–13.96)	<0.001	20.47 (10.20–41.07)	<0.001	1.27 (1.20–1.35)	<0.001
Multivariate adjusted	Reference	1.01 (0.21–-4.83)	0.983	6.95 (3.43–14.06)	<0.001	23.65 (11.45–48.87)	<0.001	1.29 (1.22–1.37)	<0.001
HGPca									
Crude	Reference	2.12 (0.71–6.30)	0.176	1.81 (0.82–3.99)	0.138	3.95 (1.80–8.69)	0.001	1.10 (1.03–1.17)	0.002
Age-adjusted	Reference	2.06 (0.69–6.15)	0.195	1.87 (0.84–4.15)	0.12	3.80 (1.70–8.45)	0.001	1.10 (1.03–1.17)	0.002
PSA-adjusted	Reference	1.49 (0.45–4.83)	0.506	1.90 (0.845–4.29)	0.12	3.38 (1.49–7.70)	0.004	1.10 (1.03–1.17)	0.003
Age and PSA- adjusted	Reference	1.50 (0.46–4.88)	0.498	1.87 (0.82–4.25)	0.131	3.45 (1.50–7.96)	0.004	1.10 (1.03–1.17)	0.003
Multivariate adjusted	Reference	1.74 (0.51–5.91)	0.37	1.81 (0.76–4.27)	0.175	3.63 (1.52–8.70)	0.004	1.10 (1.02–1.17)	0.005
